# Economic and Health Impacts Associated with a *Salmonella* Typhimurium Drinking Water Outbreak−Alamosa, CO, 2008

**DOI:** 10.1371/journal.pone.0057439

**Published:** 2013-03-18

**Authors:** Elizabeth Ailes, Philip Budge, Manjunath Shankar, Sarah Collier, William Brinton, Alicia Cronquist, Melissa Chen, Andrew Thornton, Michael J. Beach, Joan M. Brunkard

**Affiliations:** 1 International Health Resources Consulting, Atlanta, Georgia, United States; 2 National Center for Emerging and Zoonotic Infectious Diseases, Centers for Disease Control and Prevention, Atlanta, Georgia, United States; 3 Regional Epidemiologist, San Luis Valley Public Health Emergency Preparedness and Response Program, Alamosa, Colorado, United States; 4 Disease Control and Environmental Epidemiology Division, Colorado Department of Public Health and Environment, Denver, Colorado, United States; The Australian National University, Australia

## Abstract

In 2008, a large *Salmonella* outbreak caused by contamination of the municipal drinking water supply occurred in Alamosa, Colorado. The objectives of this assessment were to determine the full economic costs associated with the outbreak and the long-term health impacts on the community of Alamosa. We conducted a postal survey of City of Alamosa (2008 population: 8,746) households and businesses, and conducted in-depth interviews with local, state, and nongovernmental agencies, and City of Alamosa healthcare facilities and schools to assess the economic and long-term health impacts of the outbreak. Twenty-one percent of household survey respondents (n = 369/1,732) reported diarrheal illness during the outbreak. Of those, 29% (n = 108) reported experiencing potential long-term health consequences. Most households (n = 699/771, 91%) reported municipal water as their main drinking water source at home before the outbreak; afterwards, only 30% (n = 233) drank unfiltered municipal tap water. The outbreak’s estimated total cost to residents and businesses of Alamosa using a Monte Carlo simulation model (10,000 iterations) was approximately $1.5 million dollars (range: $196,677–$6,002,879), and rose to $2.6 million dollars (range: $1,123,471–$7,792,973) with the inclusion of outbreak response costs to local, state and nongovernmental agencies and City of Alamosa healthcare facilities and schools. This investigation documents the significant economic and health impacts associated with waterborne disease outbreaks and highlights the potential for loss of trust in public water systems following such outbreaks.

## Introduction

Community-wide outbreaks associated with public drinking water systems are rare in the United States since drinking water regulations were implemented by the Environmental Protection Agency (EPA), beginning in 1974 with the Safe Drinking Water Act (SDWA) [1], [2], [3]. However, in 2008 a large community-wide outbreak occurred in Alamosa, Colorado caused by contamination of the town’s unchlorinated municipal drinking water supply with *Salmonella* serotype *Typhimurium.*

Alamosa is a small municipality of approximately 8,800 residents situated between two mountain ranges in the San Luis Valley of south-central Colorado [4]. Prior to the outbreak, the City’s municipal water was supplied by seven artesian wells and was not chlorinated [5]. On March 14, 2008, the Alamosa County Nursing Service was notified of three culture-confirmed cases of *S.* Typhimurium among residents of Alamosa, including two cases in infants. An epidemiologic investigation conducted by local and state public health authorities identified the city’s municipal drinking water as the source of the outbreak [5]. From March 19−April 11, 2008, Alamosa water was deemed unsafe to drink and residents were under various water advisories. After April 11th, all areas of the water system had been hyperchlorinated and all drinking water restrictions lifted.

As a result of the outbreak, 434 cases, including 124 laboratory-confirmed cases, 20 hospitalizations, and one death were reported; a telephone survey conducted by the Colorado Department of Public Health and Environment (CDPHE) at the time of the outbreak indicated that an estimated 1,300 persons became ill (CDPHE, unpublished data). Anecdotal reports of subsequent complications due to *Salmonella* infections, such as anal abscesses and adverse pregnancy outcomes, were received by local public health authorities (W. Brinton, personal communication).

An extensive investigation conducted at the time of the outbreak involved multiple agencies. Water supply interruptions necessitated a large-scale response from local, state, and federal agencies, including the Colorado National Guard, and volunteer agencies. The economic burden to the community was thought to be significant due to business and school closures, missed work to care for ill family members, and the costs of obtaining potable water and other supplies. Because of the scope and extent of the outbreak and response, CDPHE and the local health department in Alamosa requested assistance from the Centers for Disease Control and Prevention (CDC) to assess the full economic and long-term health impacts on the community of Alamosa.

## Methods

### Ethics Statement

This data collection was judged by officials at CDC to be non-research public health practice, and therefore was not subject to Institutional Review Board (IRB) review. Nevertheless, written informed consent was obtained from all participants, and participants were given the option to refuse specific questions or to decline responding to the surveys.

### Data Collection

We conducted a community-wide household survey to assess the health and economic impacts of the outbreak; a business survey to assess the costs incurred by businesses; school surveys to document closures and costs; a review of billing data from two local health care systems to assess direct healthcare expenditures; and interviews with local and state governmental and non-governmental agencies to document costs related to the emergency response efforts.

### Household Survey

In October 2009, we sent or hand-delivered a survey to all households that received a water bill from the City of Alamosa (as of September 2009) and surveys were returned via postal mail to CDC. Survey questions covered topics such as residents’ drinking water source before and after the outbreak, alternate water sources used during the outbreak, household illness during the outbreak, including potential long-term health consequences (e.g., joint, skin, urinary tract, eye, or other problems occurring within one month following diarrheal onset), and other demographic and household characteristics. Households were also asked to report economic costs associated with the outbreak, including costs associated with illness (e.g., over the counter medicine and out-of pocket costs for prescription medications, doctor’s visits and hospitalization), caring for ill family members, securing alternate water sources (bottled water or water filters). To calculate indirect cost of illness, ill household members and caretakers were also asked to provide information on their occupation and daily wage (see Table S1 in File S1 for more information). Some questions were posed at the household-level (e.g., costs for purchase of bottled water) while others were reported for each member of the household (e.g. symptoms, occupation, and demographic characteristics). For our analysis, in order to be consistent with the case definition used during the outbreak, a case was defined as anyone who reported diarrhea (≥2 loose stools during a 24 hour period) during the outbreak. An affected household was any household with ≥1 person who experienced diarrhea during the outbreak.

### Business Survey

To describe how the outbreak impacted local businesses financially, in October 2009 we sent a survey to all businesses inspected by CDPHE (N = 128), including retail food establishments (restaurants, hotels, nursing homes, and child care centers), and other businesses (N = 54) potentially affected by the water shortages (grocery stores, beauty salons, dentists, and animal clinics). Contact information was provided by CDPHE, or obtained through a telephone directory and internet searches. Businesses were asked how the outbreak affected their business, including whether the business had to close, lay off workers, and whether the business had to buy additional water or ice, lost or gained money overall, and if the business ever regained pre-outbreak levels. To encourage businesses to respond and protect confidentiality, we did not ask for the business name or address on the survey. Five of the 128 CDPHE-inspected businesses were located outside of Alamosa but had clientele likely to be comprised of Alamosa residents.

### Interviews with Governmental and Non-governmental Agencies, Healthcare Facilities, and Schools

We interviewed via telephone or in-person staff from the City of Alamosa, Alamosa County Nursing Service, and CDPHE and the local chapter of the American Red Cross to ascertain estimates of the direct and indirect cost of the outbreak response to local and state governmental and non-governmental agencies. Respondents provided information on the cost of the response (e.g., lodging and meals for staff, truck rentals, etc.), the number of staff and their aggregate labor hours spent responding to the outbreak. We also interviewed Alamosa health care providers, including a hospital, medical practices, nursing homes, and assisted living facilities to assess the outbreak impact and to request billing data to supplement cost estimates from the household surveys (see Supporting Information and Table S2 in File S1for more information). To determine the effects of the outbreak on educational institutions, we interviewed representatives from each of Alamosa’s two public colleges, two private schools, and its public school district. School representatives were asked about the types of additional costs incurred because of the outbreak, including purchasing bottled water or hand sanitizer, paying for employee overtime, and costs for make-up days.

### Analysis

All survey data were entered into a Microsoft Access 2007 database and descriptive analyses were conducted using SAS v. 9.2 (Cary, NC). We compared survey respondents’ characteristics (sex, age, race/ethnicity and income) to the characteristics of the 2008 City of Alamosa population [6] using chi-square tests. All p-values were two-sided and the level of significance was 0.05.

For our cost estimates, we took a societal perspective and defined costs as expenses which would not be incurred if the outbreak had not occurred. Since almost all costs were incurred in 2008 and 2009, we did not apply a discount rate. No capital costs (materials with more than a 5 year useful life) were incurred. All the costs were recorded in 2008 U.S. dollars.

We built a Monte Carlo simulation model using @Risk software (Palisade Corporation, NY) to extrapolate the costs to the city of Alamosa. The model used the following formula (see Table S3 and Figure S1 in File S1 for more information):

**Total cost** = Number of individuals/households in Alamosa in 2008 (from census data)×Proportion of respondents who experienced a cost (from household survey) X.

Cost distribution of the given cost (from household survey).

We assumed that the proportion of respondents from the survey who experienced a given cost was the same proportion in the community who would have experienced the costs. We used the costs reported by all individuals/households in the survey to generate the cost distribution to fit the data for the model. The details of these cost distributions are given in the Supporting Information (Table S3 in File S1). Using Monte Carlo simulation (10,000 iterations), we then extrapolated the costs to the city of Alamosa and the model’s results are presented as the total cost (the median of the 10,000 iterations) and range (representing the 5^th^ to 95^th^ percentile of the 10,000 iterations). Additional methodological details about the model, methods for direct and indirect cost calculations, and extrapolation methods, are provided in the Supporting Information (see File S1).

## Results

### Household Survey

**Figure 1:**
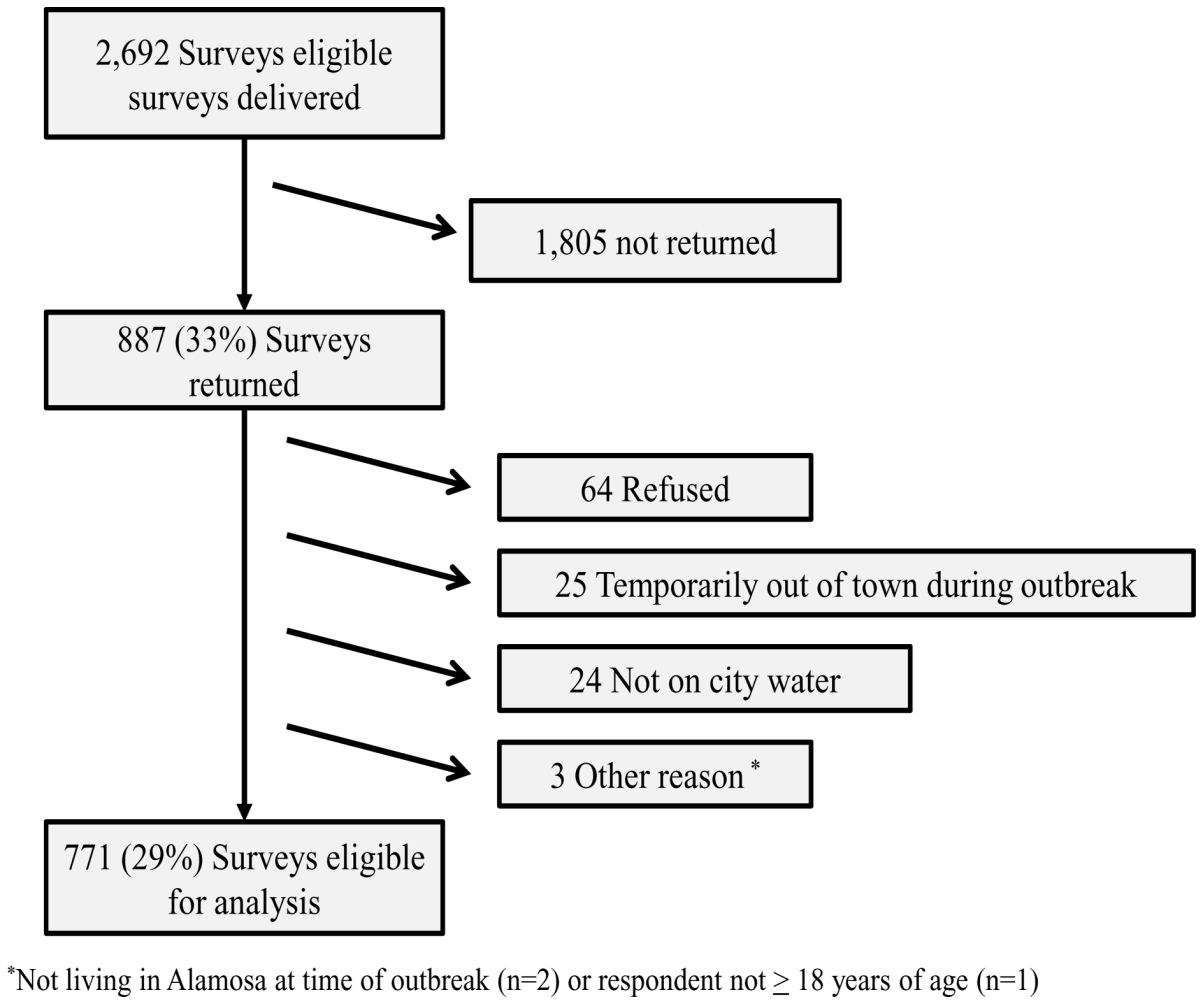
Household survey response rate.

The community survey was distributed to all households that received municipal drinking water (N = 2,692). After excluding non-responders and ineligible responses (refusal, out of town during the outbreak, not on city water, or other reasons) 29% (n = 771) of households, representing 1,732 persons, returned surveys eligible for analysis (Figure 1). The median number of persons per household was two (range: 1–8), and the surveys analyzed represented approximately 20% of the City of Alamosa’s 2008 population [4]. The majority (n = 458, 59%) of survey respondents were female; the median age of respondents was 57 years (range: 18–99). Half (n = 391, 51%) of the respondents identified themselves as white and 31% (n = 240) were of Hispanic ethnicity; 8% (n = 63) did not provide race or ethnicity information. Of the 620 (80%) households that reported income information, 14% (n = 89) made <$13,000, 18% (n = 111) $13,000−$25,000, 24% (n = 150) $25,000−$45,000, 21% (n = 130) $45,000−$75,000, and 23% (n = 140) >$75,000. Compared to the population of the City of Alamosa, our survey respondents tended to be older, more likely to be female (p<0.001), less likely to be of Hispanic ethnicity (p<0.001), and moderately more affluent (p<0.001) [6] (Table S4 in File S1).

#### Illness associated with the Outbreak

Approximately one-third (242/771, 31%) of households, and 21% (369/1732) of individual respondents, reported diarrheal illness during the outbreak. Fifty-seven percent (n = 187/329) of ill persons were female (including four who were pregnant); the median age of ill persons was 37 years (range: 0–98 years). By definition, all ill persons experienced diarrhea out of which 30% (n = 110) reported bloody diarrhea. The median duration of illness was four days (range: 1–60 days). In total, survey respondents (n = 350) were sick for 2,341 person-days. Most (n = 194, 80%) of the Alamosa households with at least one ill person reported buying medicine or other items because of their illness (Table 1); 31% of households (n = 75) with at least one ill person sought care, and close to half of those who sought care reported receiving a prescription (n = 32, 43%) or having a diagnostic test performed (n = 37, 49%) (Table 1).

**Table 1:** Costs to City of Alamosa households associated with an outbreak of salmonellosis, Alamosa, Colorado 2008.

		Household Survey				City of Alamosa (2008)	
		(n = 1,732 individuals, 771 households)				(N = 8,746 persons, 3,302 households)	
			Cost($)				
			Reported in survey*			Simulation model†	
	n(%)incurred cost	n(%)reported cost*	Mean	Median	Total	Total	(range)
**Outbreak-related expenses (n = 771 households)**							
Bought bottled water	657(85%)	523(68%)	$87	$50	$45,530	$135,781	($30,498–$604,183)
Bought water filtration system	202(26%)						
Installation of filter	202(26%)	195(25%)	$180	$70	$35,084	$83,536	($7,760–$336,936)
Maintenance of filter	202(26%)	168(22%)	$121	$60	$20,343	$53,715	($3,974–$232,136)
Stay overnight somewhere else	123(16%)	90(12%)	$362	$233	$32,549	$113,266	($31,749–$403,950)
***Subtotal***					***$133,506***	***$386,298***	***($73,981–$1,577,205)***
**Direct out-of-pocket health care costs (n = 242 households reporting at least one ill person with diarrhea)**							
Bought items or received medical treatment	194(80%)	(0%)					
Bought nonprescription medicine	168(69%)	162(67%)	$40	$25	$6,483	$18,220	($4,617–$71,847)
Bought other things (e.g., Gatorade or diapers)	120(50%)	116(48%)	$44	$25	$5,102	$13,765	($4,127–$45,893)
Went to doctor or a clinic	75(31%)	64(26%)	$80	$43	$5,116	$12,653	($936–$54,660)
Received a prescription	32(43%)	28(44%)	$17	$13	$463	$1,850	($397–$5,193)
Had diagnostic tests (e.g., blood or stool test)	37(49%)	35(55%)	$96	$30	$3,355	$5,508	($408–$23,801)
Went to hospital/emergency room	31(13%)	21(9%)	$320	$40	$6,725	$10,603	($784–$45,825)
***Subtotal***					***$27,244***	***$62,599***	***($11,269–$247,219)***
**Indirect cost of acute illness**							
**Ill persons (n = 369)**							
Work full-time	156(42%)	87(24%)	$430	$300	$2,917	$183,644	($45,663–$480,473)
Work part-time	102(28%)	8(2%)	$215	$150	$37,889	$47,083	($11,716–$123,194)
Non-worker	111(30%)	(0%)	$0	$0	$0	$0	(n/a)
**Caretakers (n = 106 caretakers)**							
Paid caretakers	11(10%)	7(7%)	$178	$110	$1,245	$4,676	(n/a)
Unpaid caretaker (work full-time)	77(73%)	59(56%)	$913	$588	$54,165	$148,173	($31,958–$686,232)
Unpaid caretaker (work part-time)	15(14%)	1(1%)	$457	$294	$640	$14,434	($3,115–$66,844)
Unpaid caretaker (non-worker)	3(3%)	(0%)	$0	$0	$0	$0	(n/a)
***Subtotal***					***$96,856***	***$398,010***	***($97,218–$1,361,419)***
**Total for Households in Alamosa**					**$257,606**	**$846,907**	**($182,468–$3,185,843)**

* Respondents who incurred cost and reported an estimate of that cost (not every respondent who reported incurring a cost provided the specific cost estimate).

† Costs extrapolated to the City of Alamosa as: total individuals/households in City of Alamosa (from census)×% incurring costs (column 3 above)×cost distribution (Table S3 in File S1) using a Monte Carlo simulation model with 10,000 iterations. Total cost derived from median of 10,000 iterations and range represents the 5^th^ to 95^th^ percentiles of the 10,000 iterations of the Monte Carlo simulation model. See main text and Supporting Information (in File S1) for details.

Twenty-nine percent (n = 108) of all ill persons reported experiencing ≥1 potential long-term health consequence of *Salmonella* infection, ranging from 14% reporting skin or joint problems to 2% reporting abscesses or more serious complications (e.g., bowel perforation, septic arthritis, or endocarditis) (Table 2). These symptoms began between four days (mean for eye problems) to seven days (mean for joint and urinary tract problems) after diarrhea onset. Among those whose symptoms had abated, symptoms lasted between two weeks (for abscesses) to four weeks (for urinary tract infections). However, 26% (n = 28) of those with long-term symptoms indicated that ≥1 symptom was still present at the time of the survey (18 months after the outbreak), ranging from 11% (for eye problems) to 39% (for joint problems) (Table 2).

**Table 2:** Potential long-term health consequences of *Salmonella* infection reported by survey respondents that were ill with diarrhea (≥2 loose stools during a 24-hour period) (n = 369) during an outbreak of salmonellosis, Alamosa, Colorado 2008.

Potential long-term health consequence	n (%)	Days after diarrhea began that problem started: mean (range)*	Duration (weeks)	
			Mean (range) time-limited duration of symptoms†	Symptoms still present at time of survey n(%)
Rash, itchiness or other skin problems	52 (14%)	5 days (0–30)	3 weeks (1–24)	11/52 (21%)
Arthritis, aching joints or other joint problems	51 (14%)	7 days (1–30)	3 weeks (0–16)	20/51 (39%)
Urinary tract problems (e.g., pain or burning during urinationor a discharge)	32 (9%)	7 days (1–30)	4 weeks (1–30)	5/32 (16%)
Eye problems such as pain or redness	19 (5%)	4 days (1–7)	2.5 weeks (1–6)	2/19 (11%)
Abscess (skin, soft tissue, anal, etc.)	6 (2%)	5 days (2–14)	2 weeks (1–3)	1/6 (17%)
Other serious complications (e.g., bowel perforation or peritonitis, septic arthritis, or endocarditis)	7 (2%)	n/a‡	n/a‡	n/a‡

* As reported by 43/52 with skin problems, 36/51 with joint problems, 27/32 with urinary tract problems, 16/19 with eye problems, and 5/6 with abscesses.

† As reported 36/52 with skin problems, by 22/51 with joint problems, 15/32 with urinary tract problems, 13/19 with eye problems, and 4/6 with abscesses.

‡ Questions were not asked.

#### Alternate water sources used during the outbreak

During the bottled water advisory, Do Not Use order, and boil water advisory, most households reported using bottled water (either purchased or donated) for drinking, cooking and brushing teeth (Table 3). Other water sources, such as boiled or treated tap water and water outside of Alamosa (e.g., at a hotel or friend’s house), were used less frequently. Despite being told to avoid using tap water except for flushing toilets during the Do Not Use order, many households continued to use tap water for at least some potable and non-potable purposes (Table 4). Half of households (55%, n = 427) reported boiling water during the 24 days of the water emergency. Eighty-five percent of households (n = 657) bought bottled water during the outbreak and spent, on average, $87 (Table 1).

**Table 3:** Number and percent of households that reported using various alternate water sources (n = 771 households)* during an outbreak of salmonellosis, Alamosa, Colorado 2008.

	Avoided activity	Bought bottled water	Used bottled/bulk water given out for free	Boiled tap water	Used water from outside Alamosa†	Used treatedtap water‡	Used un-boiledtap water	Used water from other source
**Drinking**	9(1%)	602(78%)	556(78%)	90(12%)	228(30%)	21(3%)	14(2%)	5(1%)
**Cooking**	33(4%)	465(60%)	531(60%)	233(30%)	238(31%)	28(4%)	42(5%)	9(1%)
**Dishwashing**	121(16%)	272(35%)	368(35%)	330(43%)	225(29%)	59(8%)	158(20%)	35(5%)
**Brushing teeth**	5(1%)	518(67%)	497(67%)	107(14%)	194(25%)	20(3%)	54(7%)	6(1%)
**Showering/bathing**	121(16%)	107(14%)	189(14%)	134(17%)	427(55%)	27(4%)	366(47%)	23(3%)

* Questions allowed for multiple options and therefore row totals do not sum to 100%.

† e.g., a friend’s house, hotel, or artesian spring.

‡ e.g., using chlorine or a filter.

**Table 4:** Number and percent of households that reported using *any* tap water for the following purposes during the various water advisories (n = 771 households) associated with an outbreak of salmonellosis, Alamosa, Colorado 2008.

	Bottled Water Advisory*	Do Not Use Order†	Boil Water Advisory‡
	(March 19–24, 2008)	(March 25–April 3, 2008)	(April 3–11, 2008)
**Drinking**	71(9%)	25(3%)	58(8%)
**Cooking**	212(28%)	73(9%)	233(30%)
**Washing dishes**	414(54%)	191(25%)	456(59%)
**Brushing teeth**	145(19%)	58(8%)	116(15%)
**Showering/Bathing**	516(67%)	256(33%)	498(65%)

* During the bottled water advisory, residents were told to use bottled water for drinking, cooking, brushing teeth, and dishwashing but that if no bottled water was available they could boil their water.

† During the Do Not Use order, while the distribution system was being hyperchlorinated, residents were told to only use their tap water for flushing toilets.

‡ During the boil water advisory, residents were told to boil their water before using it for drinking, cooking, or brushing teeth.

#### Drinking water impact

**Figure 2:**
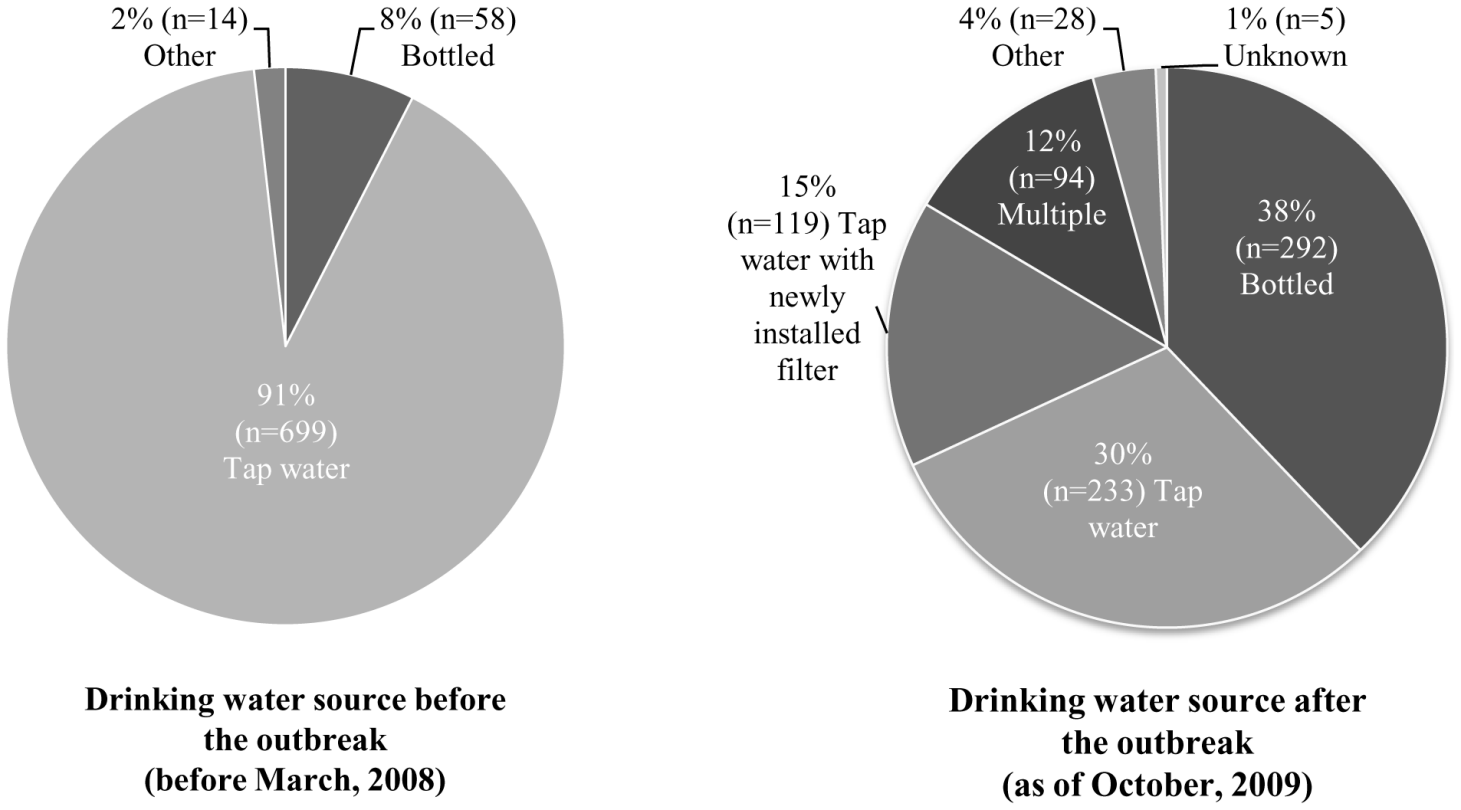
Change in water source after the outbreak (n = 771 households) following an outbreak of salmonellosis, Alamosa, Colorado 2008.

Most households (n = 699, 91%) reported municipal tap water as their main drinking water source at home prior to the outbreak (Figure 2). Eighteen months after the outbreak, the main drinking water sources among Alamosa survey respondents were: bottled water (38%, n = 292/771), municipal water (30%, n = 233), municipal water with a new filter installed (15%, n = 119), multiple (12%, n = 94), or other sources (4%, n = 28) (Figure 2). Taste, safety, and smell were the main reasons cited for switching from municipal tap water to bottled water or adding a new water filter after the outbreak (Table 5).

**Table 5:** Reasons households cited for switching from tap water as a main drinking water source to bottled water as a main drinking water source (n = 249) or installing a new water filter (n = 114) after an outbreak of salmonellosis, Alamosa, Colorado 2008.

	Switched from tap to bottled water (n = 249)*	Added a new filter (n = 114)*
**Taste**	134(55%)	75(66%)
**Safety**	110(45%)	42(37%)
**Smell**	60(25%)	35(31%)
**Color or other reason**	29(12%)	16(14%)

* Categories are not mutually exclusive so percentages can sum to >100%.

#### Household economic impacts

Outbreak-related costs for City of Alamosa residents totaled an estimated $846,907 (range: $182,468–$3,185,843) (Table 1). The estimated total cost associated with the purchase of supplemental bottled water, installing and maintaining new water filters, and paying to stay overnight elsewhere was $386,298. Direct costs associated with illness (e.g., purchasing over the counter medicines or other items, doctors’ visits, prescription medications, diagnostic tests, and hospital stays) were estimated to total $62,599. Additional indirect costs of acute diarrheal illness and costs associated with paying for, or lost productivity due to, care of ill persons and children were estimated to total $398,010 (Table 1).

### Business Survey

We distributed 177 surveys to establishments inside the City of Alamosa and 5 to businesses located outside the City of Alamosa that primarily served or employed Alamosa residents. Of the 182 surveys, 21 (12%) were undeliverable and 50 (50/161, 31%) were returned. The following surveys were excluded: three because the business was not open during the outbreak and one because it was a city jail. Forty-six (46/161, 29%) surveys were eligible for analysis: 41 surveys were included in the primary analysis and an additional five surveys were analyzed separately because the business was either located outside Alamosa or not connected to municipal water.

The 41 businesses located in Alamosa and connected to municipal water included retail stores (n = 8, 20%), restaurants or other food service establishments (n = 6, 15%), beauty salons or barber shops (n = 6, 15%), child care centers (n = 4, 10%), nursing homes or long-term care facilities (n = 3, 7%), and other types of businesses (n = 10, 24%); two (5%) businesses did not specify the type of establishment. One-third (14/41, 34%) of responding businesses closed during the outbreak (mean length of business closure: 8.4 days). Approximately half of businesses reported losing money due to the outbreak, with a median loss of $8,750 (range: $400–$200,000) (Table 6). One business outside of Alamosa reported a total loss of $13,967. Four businesses (10%) never returned to pre-outbreak financial levels, including one business that closed permanently due to the outbreak. The total estimated cost of the outbreak extrapolated to the City of Alamosa businesses that are known to have used municipal water (n = 156) was $625,561 (range: $14,209–$2,817,036) (Table 6).

**Table 6:** Outbreak-associated costs to City of Alamosa businesses associated with an outbreak of salmonellosis, Alamosa, Colorado 2008.

	Business Survey (n = 46)			Extrapolated to Sample ofCity of Alamosa Businesses (N = 156)	
		Reported in survey		Estimated using simulation model*	
	n(%)	Mean	Median	Total (range)	
**Businesses inside Alamosa and on city water (n = 41)**					
Lost money	22(54%)	$35,306	$8,750	**$625,561**	**($14,209**–**$2,817,036)**
No change	17(41%)				
Did better because of the outbreak	2(5%)				
**Businesses outside Alamosa or not on city water (n = 5)**					
Lost money	1(20%)	$13,967	$13,967	**$13,967**	**n/a**
No change	3(60%)				
Did better because of the outbreak	1(20%)				
**Total**				**639,528**	**($28,176**–**$2,831,003)**

* Costs extrapolated to the City of Alamosa as: total businesses on municipal water (N = 156)×% incurring costs (column 3 above)×cost distribution (Table S3 in File S1) using a Monte Carlo simulation model with 10,000 iterations. Total cost derived from median of 10,000 iterations and range represents the 5^th^ to 95^th^ percentiles of the 10,000 iterations of the Monte Carlo simulation model. See main text and Supporting Information (in File S1) for details.

### Governmental and Nongovernmental Agencies, Healthcare Facilities, and Schools

Outbreak response cost estimates to local, regional, and state governmental and volunteer organizations totaled $823,314 (Table 7). This estimate primarily included the governmental response costs for the National Guard, incident management teams, and personnel, and other costs (such as transportation, supplies, lodging, etc.) at state, county, and local levels. The volunteer response, coordinated primarily by the American Red Cross, lasted 21 days, involved 1,035 people who contributed over 5,289 hours to the effort, and totaled an estimated $80,710 (Table 7).

**Table 7:** Outbreak response costs associated with an outbreak of salmonellosis, Alamosa, Colorado 2008.

	Personnel	Other*	Total
**Governmental organizations**			
Federal Government	$19,040†		$19,040
State of Colorado	$215,925	$316,449‡	$532,374
Alamosa County	$52,817	$7,582	$60,399
City of Alamosa	$50,872	$19,023	$69,895
**Non-governmental organizations**			
Volunteer organizations	$40,135#	$40,575	$80,710
Other organizations	$60,896†		$60,896
**Total**	**$439,685**	**$383,629**	**$823,314**

* Includes transportation, supplies, lodging, etc.

† As captured by City of Alamosa record-keeping.

‡ Includes expenses covered by the state disaster fund (e.g., National Guard, incident management teams); these were all included in the “Other” category because personnel costs were not reported separately from other expenses.

# Includes $7,920 for staff overtime (3 persons and a total of 750 hours of overtime) and $32,215 in estimated indirect costs associated with volunteer time (4,589 volunteer hours estimated at Colorado minimum wage rate of $7.02/hour).

Most health care providers reported significant expenses in securing and providing clean water (for drinking, bathing, housekeeping and other uses) or disposable supplies. However, these costs could not be estimated because most could not retrospectively itemize these expenses. Only one local hospital was able to provide billing records for outbreak-related care it provided to 104 of the 124 laboratory-confirmed cases. The estimated total cost of health insurance payments for Alamosa City residents that sought health care was $244,985 (range: $65,615–$928,915) (Table 8). The five public and private schools and colleges were closed for, on average, 2 days (range: 0 to 5) due to the outbreak. However, anecdotally we learned that the outbreak occurred during spring break for most of the five public and private schools and colleges in Alamosa, which helped minimize the impact of school closures. Only two schools reported any substantial financial impact, which totaled $23,898 (Table 9). These costs were related to paying overtime and purchasing bottled water and other items.

**Table 8:** Health insurance payments for Alamosa City residents that sought healthcare during an outbreak of salmonellosis, Alamosa, Colorado 2008.

	Household Survey	Hospital A Cost Estimates			Extrapolated to City of Alamosa					
	(n = 1,732)	(n = 104 culture-confirmed casesand 139 separate healthcare visits)				(N = 8,746)				
						Cost ($)‡				
	n(%)	n(%)	Mean	Median	N(%)†	Total (range)				
Ill Persons	369(21%)				1,423(16%)					
Sought care	107(29%)				413(29%)					
Clinic/doctors’ office*	76(71%)	67(48%)	$129	$93	293(71%)	**$26,275**		**($6,430**–**$107,324)**		
Emergency department	22(21%)	67(48%)	$693	$390	85(21%)	**$36,600**		**($7,351**–**$182,111)**		
Hospitalized	9(8%)	5(4%)	$7,011	$3,159	35(8%)	**$182,110**		**($51,834**–**$639,480)**		
**Total**		**139**				**$244,985**		**($65,615**–**$928,915)**		

* Because data were obtained from the hospital only, clinic/doctor’s office visit costs only include laboratory but not physicians’ fees.

† We have removed the background rate of diarrhea in the population (5%) to get the percent of illness due to outbreak (21%−5% = 16%). The number of ill persons was the denominator for subsequent proportions who incurred the costs (e.g., 29%,71%, 21% and 8%).

‡ Costs extrapolated to the City of Alamosa as: total population (N = 8,746)×% incurring costs (column 3 above)×cost distribution (Table S3 in File S1) using a Monte Carlo simulation model with 10,000 iterations. Total cost derived from median of 10,000 iterations and range represents the 5^th^ to 95^th^ percentiles of the 10,000 iterations of the Monte Carlo simulation model. See main text and Supporting Information (in File S1) for details.

**Table 9:** Total costs associated with an outbreak of salmonellosis, Alamosa, Colorado 2008.

	Total	(range)	% of total
**City of Alamosa households**	**$846,907**	**($182,468–$3,185,843)**	**32.8%**
Outbreak-related expenses	$386,298	($73,981–$1,577,205)	
Direct out-of-pocket health care costs	$62,599	($11,269–$247,219)	
Indirect costs of acute illness and caretaking	$398,010	($97,218–$1,361,419)	
**Alamosa businesses**	**$625,561**	**($14,209–$2,817,036)**	**24.3%**
**Businesses outside the City of Alamosa**	**$13,967**	**n/a**	**0.5%**
**Governmental organizations**	**$681,708**	**n/a**	**26.4%**
Federal Government	$19,040	n/a	
State of Colorado	$532,374	n/a	
Alamosa County	$60,399	n/a	
City of Alamosa	$69,895	n/a	
**Non-governmental organizations**	**$141,606**	**n/a**	**5.5%**
Volunteer organizations	$80,710	n/a	
Other	$60,896	n/a	
**Health insurance payments**	**$244,985**	**($65,615–$928,915)**	**9.5%**
**School and colleges**	**$23,898**	**n/a**	**0.9%**
***Grand Total***	**$2,578,632**	**($1,123,471–$7,792,973)**	**100%**

Details provided in Tables 1, 6 & 8. Extrapolation done using Monte Carlo simulation model. See main text and Supporting Information in File S1 for details.

### Total Costs

The total estimated economic impact of the outbreak, including costs to City of Alamosa residents, businesses, schools, and healthcare facilities and the governmental and non-governmental outbreak response was approximately $2.6 million (range: $1.1 million–$7.8 million dollars) (Table 9). The largest contributors (32.8%) to this cost were direct and indirect costs for City of Alamosa residents, followed by the cost of the outbreak response to governmental organizations (26.4%), and costs to Alamosa businesses (24.3%).

## Discussion

Since passage of the Safe Drinking Water Act and its amendments by EPA [1], [2], [3], community-wide drinking water outbreaks in the U.S. are rare [7]. The *Salmonella* outbreak that occurred in Alamosa, Colorado in 2008 was one of the largest drinking water-associated outbreaks reported in the U.S. since the 1993 *Cryptosporidium* outbreak in Milwaukee, which sickened an estimated 400,000 people [8]. The estimated economic impact of the Alamosa outbreak totaled over $2.6 million. An unanticipated consequence of the outbreak was the loss of trust in the public water system after the outbreak.

Despite our comprehensive approach, this outbreak cost estimate is lower than previous epidemiologic studies of outbreaks in public water systems, perhaps due to our conservative methodological approach and the differences in the size of the affected population or duration of the outbreak. Harrington et al. estimated the economic impact of a 1984 waterborne outbreak of giardiasis in a Pennsylvania county at $18.2–133.3 million (in 2008 dollars) and did not include the cost of the outbreak response or the impact on local businesses [9]. The outbreak occurred in a community of 25,000 households, resulted in 370 cases of giardiasis and a boil water advisory that lasted at least 99 days (270 days for half of those affected). Corso et al. estimated that the massive 1993 cryptosporidiosis outbreak in Milwaukee, Wisconsin (population ∼ 1.6 million) affecting 403,000 individuals cost an estimated $143.4 million (in 2008 dollars) in direct healthcare expenditures and productivity losses [10]; the cost of using an alternate water source during the outbreak, costs to local businesses, and the cost of the outbreak response were not included. Despite these differences, the limited number of cost analyses for waterborne outbreaks underscores the need to conduct these analyses for future outbreak investigations and the utility of including longer follow-up investigations to capture long-term costs.

In our assessment, 31% of households and 21% of survey respondents became ill during the outbreak. Approximately one-third of those who became sick reported a potential long-term health consequence following their diarrheal illness and, of those, 26% were still experiencing symptoms 18 months after the outbreak. Because all symptoms were based on self-report, and may have been coincidental to, rather than caused by the *Salmonella* outbreak, we included only symptoms that began within 30 days of diarrheal illness. In addition, similar frequencies of post-*Salmonella* infection joint pain [11], [12] and other symptoms [13] have been observed in previous studies, including a Canadian study that found that such symptoms can persist for three years post-infection [14]. Although they were rare, we also found that 2% of cases experienced a more serious complication of infection such as bowel perforation, peritonitis, septic arthritis, or endocarditis, complications from *Salmonella* infection that have been reported elsewhere [15]. Unfortunately, we were unable to estimate the direct and indirect costs associated with these long-term sequelae. However, the time and costs associated with these are likely to have been substantial. For instance, patients with arthritis and other rheumatologic conditions had average annual medical care expenditures of $1,891 and earned $1,590 less than individuals without these conditions in 2003 [16]. Additionally, urinary tract infections cost an estimated $1.6 billion per year in 1994 in direct and indirect costs [17].

Over 90% of households reported that municipal water was their main drinking water source at home prior to the outbreak. After the outbreak, 38% of respondents mainly drank bottled water and only 30% of households continued to primarily drink tap water; an additional 15% purchased a new filter or filtration system. The purchase of bottled water and installation and maintenance of filters cost City of Alamosa residents approximately $273,000 during the outbreak. Almost half (45%) of survey respondents cited safety concerns as a reason for switching from tap to bottled water. This lack of trust was also apparent in survey participants’ comments, such as: “I will never again fully trust the system or drink any tap water without some concern…” and “I still don’t feel safe drinking or cooking with the city water… I have spent a lot of money buying bottled water.”

The economic impact of the outbreak on the sample of businesses was one of the largest expenses, totaling $626,000 and accounting for 24% of the total outbreak costs. Approximately half of businesses that responded indicated that they lost money and approximately one-third had to close temporarily during the outbreak. Only 60% reported ever returning to pre-outbreak financial levels, including one that noted that “it took 2–3 months to get back to previous levels.” Because the survey was sent 18 months after the outbreak, it could have failed to reach businesses that might have been forced to close because of the outbreak. Household survey responses corroborated this; one respondent noted that “we couldn’t pay our mortgage [and] lost our restaurant. We now both work for someone else for not as much pay. We had our restaurant for 25 years.”

This assessment was subject to several limitations. First, our outbreak cost estimate is likely an underestimate. It does not include health care costs for individuals who sought care outside of Alamosa (either because some ill individuals may not have responded to the survey or because we were only able to obtain hospital-associated costs from one local hospital). We also were unable to assign an estimate for the one death associated with the outbreak. Alamosa is the geographic and commercial center of the San Luis Valley, and many people from surrounding areas work and dine in Alamosa but the survey did not capture business-related costs or health impact for people who live outside of Alamosa or for businesses that either did not receive a survey or did not respond. Additionally, outbreak response costs incurred by the federal government and by local organizations or municipalities outside the City of Alamosa that contributed to the outbreak response are likely incomplete. Household survey respondents also mentioned various costs not covered in the questionnaire, such as the cost of gas, disposable plates/utensils, or pet care associated with the outbreak. Additionally, direct and indirect costs associated with the long-term health consequences of *Salmonella* infection were not assessed. Outbreak-associated costs were also limited to those incurred during the outbreak, even though it is likely that many costs have been incurred since the outbreak.

Second, we assumed that the survey respondents were a representative sample of the City of Alamosa population, yet our survey respondents differed by age, sex, ethnicity, and socioeconomic status [6]. In addition, our survey was conducted 18 months after the outbreak, and persons who responded to the survey may have been more likely to be sick during the outbreak and therefore to respond to the survey, although the attack rate estimated from our household survey is similar to the estimate found in a survey of Alamosa residents immediately after the outbreak (CDPHE, unpublished data). Third, our indirect cost estimates were based on assumptions (see Supporting Information in File S1 for details) about the value of caretakers’ and ill people’s time. However, the wages reported in our survey were similar to that reported for the Colorado non-Metropolitan Statistical Area that includes Alamosa [18]. Finally, our relatively low response rate (∼ 30%), although similar to that of other mailed surveys [19], may mean that the results of the household and business surveys are not reflective of the experiences of all City of Alamosa households and businesses.

The likely source of the outbreak was determined to be animal contamination of a storage tank that had numerous cracks and entry points [5]. This outbreak highlights the critical importance of robust inspection of public drinking water storage facilities, identification of system deficiencies during required sanitary surveys, and maintaining staffing and resources for adequate follow-up for any deficiencies identified. Although it is now being chlorinated, the City of Alamosa’s water prior to the outbreak was derived from an unchlorinated ground water source. The recently promulgated Ground Water Rule (GWR) [20], [21] requires most community water systems to complete initial sanitary surveys by 2012. Once fully implemented, it should help reduce the risk for similar outbreaks in the future. Nevertheless, a deficiency in the distribution system (i.e., storage tank contamination) was the primary cause of this outbreak. Maintaining the integrity of the nation’s drinking water systems is a fundamental safeguard to protecting public health and preventing economic damage from waterborne disease outbreaks and should be a top public policy imperative.

## Supporting Information

File S1 (DOCX)
