# Supplementary material for: Economic and Health Impacts Associated with a Salmonella Typhimurium Drinking Water Outbreak−Alamosa, CO, 2008
Source: PLoS One. 2013 Mar 18;8(3):e57439. doi: 10.1371/journal.pone.0057439 (PMC3601119; doi:10.1371/journal.pone.0057439)
Supplement: File S1 — (DOCX) [file pone.0057439.s001.docx]

**Supporting Information File S1: Cost Analysis of the 2008 Salmonellosis Outbreak, Alamosa, CO**

**Approach:** We collected information on outbreak-associated costs at the individual, household and community levels. We took a societal perspective and defined costs as expenses which would not be incurred if the outbreak had not occurred. Since almost all costs were incurred in 2008 and 2009, we did not apply a discount rate. No capital costs (materials with more than a 5 year useful life) were incurred. All the costs were recorded in 2008 U.S. dollars.

**Costs**

*Outbreak-Related Expenses to Households*

Households bought bottled water (in addition to the water that was given out for free), water filters during and after the outbreak, and temporarily took up residences outside of Alamosa during the outbreak. Information on these costs was obtained from the household survey.

*Direct Out-of-Pocket Health Care Costs*

The household survey captured the proportion of households and the amount each household incurred for direct out-of-pockets medical costs, including: (i) the cost of nonprescription medications or other items bought due to illness during the outbreak; (ii) the out-of-pocket cost of a clinic or doctor’s visit; (iii) the out-of-pocket costs associated with diagnostic tests and prescription medications; and (iv) the out-of-pocket cost associated with a visit to an emergency room or hospital.

From the household survey, we obtained the proportion of ill persons who sought care for diarrheal illness and categorized them by their heath care visit type (clinic/doctors' office, emergency department, or hospitalization).

*Indirect Costs of Acute Illness and Caretaking*

In addition to the direct costs of illness, Alamosa residents incurred indirect costs from the outbreak and response. Residents reported missing work because of illness or because they were taking care of ill persons or children home due to school closure. These indirect costs were calculated as a function of a person’s reported daily wage rate and the number of days ill or spent caretaking. Ill persons and caretakers were classified into full-time (those who would be working if not sick or caretaking), part-time (persons who reported that they were retired or would be staying at home or on vacation during the time of their illness or caretaking) and non-workers (persons who would have been going to school or who had an unknown occupation) (see table S1). In order to account for days where a person might have been ill but still able to do his or her normal daily activities, we calculated days of lost productivity. These were either the total days that the person was ill or the number of days the person was completely unable to do normal activities plus half of the days s/he was somewhat unable to do normal activities, if this was less than the number of days ill. For those who reported a greater number of non-productive days than days ill, we used the lower estimate of the number of days ill. For caretakers, the number of days of lost productivity was the number of days that the caretaker reported taking care of ill people plus the number of days that she or he reported taking care of children home from school due to the outbreak.

The distribution of the indirect costs was obtained by multiplying an individual’s daily wage by number of non-productive or caretaking days. We assumed that a full-time worker had the same indirect cost distribution (irrespective of taking sick day or not) whereas a part-time worker had a distribution of 50% of the indirect costs. The Human Capital approach undervalues household production; for instance, Grosse (2003) [1] allocated lower wage rates for household services (65-75% of those who work). Therefore, in order to be conservative, we assumed that part-time workers’ indirect cost distribution was 50% of the indirect cost distribution of a full-time worker. As most of the outbreak occurred during spring break, no cost was assigned to persons who would have been attending school. Those with unknown occupations were assigned values based on their age: people with unknown occupations who were > 18 were assigned the same indirect costs as the part-time workers while those < 18 years of age or with missing age were assumed not to incur any costs.

*Costs for City of Alamosa Businesses*

From the business survey we obtained the proportion of businesses inside Alamosa and on city water that reported losing money as a result of the outbreak as well as their total outbreak-associated costs.

*Costs Associated with Volunteers*

Volunteers from American Red Cross aided in the outbreak response. We used the 2008 minimum wage of Colorado ($7.02/hour) [2] to assign an opportunity cost for time contributed by volunteers. The cost for volunteers was estimated by multiplying the 2008 minimum wage of Colorado by the total number of volunteer hours reported by the American Red Cross.

*Health Insurance Payment Costs*

To get additional information, including health insurance payments, on costs for ill persons who sought health care, we obtained billing records of treatment costs (costs reimbursed by health insurance companies) from a local hospital where 104 of the 124 culture-confirmed outbreak-associated cases were treated. We obtained billing and payment data for hospitalizations, emergency department visits, and laboratory testing charges for specimens obtained from clinic/doctors' office visits. We categorized the reimbursed costs according to the health care visit type and used the summary measures (mean and standard deviation) for modeling. Because cost data were obtained from the hospital only, clinic/doctors’ office visit costs included laboratory fees but not physicians' fees.

**Extrapolation of Survey Data to City of Alamosa Population**

The population of the City of Alamosa at the time of the outbreak (in 2008) was 8,746 persons [3] or 3,302 households [4]. We estimated the total number of ill persons and households in the City of Alamosa using the proportion of people (21%) and households (31%) that reported illness from our household survey and then subtracted the background rate of diarrhea (5%) obtained from a population-based study [5]. Therefore, we estimated that 1,426 City of Alamosa residents in 859 households were ill with diarrhea during the outbreak. Additionally, based on the frequency of health care use reported among individuals in the household survey (21%) and the proportion of those who sought health care and reported being hospitalized (8%), we estimated that 413 City of Alamosa residents sought health care (n=378 as outpatients and 35 as hospitalized patients) (Table S2). For Alamosa businesses, we were only able to extrapolate to the total number of eligible businesses in our convenience sample (n=156), which was calculated by removing the number of undeliverable surveys (n=21) and surveys from businesses outside of Alamosa (n=5) from the total number of surveys we distributed (n=182).

Initial exploratory data analyses using histograms and summary descriptive statistics revealed that all the costs were positively skewed and had a high variance. For extrapolation from the survey to the City of Alamosa, we built a Monte Carlo simulation model using @Risk 5.5 software (Palisade Corporation, New York). The model used the following formula

**Total cost** = Number of individuals/households in Alamosa in 2008 (from Census data) X Proportion of respondents who experienced a cost (from household survey) X

Cost distribution of the given cost (from household survey)

A schematic of the model is given in Figure S1. Input distributions of primary cost data were determined using the ‘Fit Distribution to Data’ function of @Risk except for outbreak-related expenses, direct out-of-pocket medical costs, and indirect costs of acute illness and caretaking. The most appropriate distribution was selected from those suggested based on chi-square statistics, relative frequencies and quantile-quantile graphs. The distributions were assigned a lower bound of 0 since all costs were positive (Table S3). For direct medical costs that were reimbursed, we assumed a log normal distribution and used the mean and standard deviation.

We then assumed that the proportion of individuals (or households) that reported incurring a specific cost represented the fraction of the population of the City of Alamosa who also incurred that specific cost. Using Monte Carlo simulation (10,000 iterations), we extrapolated the costs to the city of Alamosa to obtain a range of total costs.

**References**

1. Grosse SD (2003) Appendix I. Productivity loss tables. In: Prevention Effectiveness, 2nd ed (Haddix AC, Teutsch SM, Corso PS., ed). New York:Oxford University Press, 255-257.
2. Colorado Department of Labor and Employment. Minimum Wage History. <http://www.colorado.gov/cs/Satellite/CDLE-LaborLaws/CDLE/1251566749488>
3. U.S. Census Bureau. 2008 Population Estimates, Alamosa City Coloardo. http://www.census.gov/popest/data/cities/totals/2009/tables/SUB-EST2009-04-08.csv.
4. U.S. Census Bureau. 2005-2009 American Community Survey 5-Year Estimates: Alamosa City, CO. <http://factfinder2.census.gov/bkmk/table/1.0/en/ACS/09_5YR/DP5YR5/1600000US0801090>
5. Jones TF, McMillian MB, Scallan E, Frenzen PD, Cronquist AB, Thomas S, et al. (2007) A population-based estimate of the substantial burden of diarrhoeal disease in the United States; FoodNet, 1996-2003. Epidemiol Infect 135: 293-301.

**TABLES**

**Table S1: Indirect cost calculations**

| **Occupation Category** | **Indirect Cost** |
| --- | --- |
| Work for Wage | Wage rate per day **X** no. of non-productive days, or  Wage rate per day **X** the no. of days ill (if non productive days > no. of days ill), or  Wage rate per day **X** no. of days spent caretaking (for caretakers) |
| Retired | 50% of those who work for wage |
| Stay at Home | 50% of those who work for wage |
| Other | 50% of those who work for wage |
| Go to School | $0 |
| Unknown | $0 |

**Table S2: Extrapolation of health insurance payments to the City of Alamosa**

|  | **Household survey** | **City of Alamosa** |
| --- | --- | --- |
|  | (n=1,732 persons) | (N=8,746 persons) |
|  | **n(%)** | **N(%)** |
| Ill Persons | 369(21%) | 1,423 |
| Sought care (doctor’s office, clinic or ER, hospital) | 107(29%) | 413 (29%) |
| Clinic/doctors' office*  Emergency department  Hospital | 76(71%)  22(21%)  9(8%) | 294(71%)  84(21%)  35(8%) |

* Because data were obtained from the hospital only, clinic/doctor's office visit costs only include laboratory but not

physicians' fees.

**Table S3: @Risk model inputs for extrapolation of costs to the City of Alamosa**

| **Cost Items** | **Distribution** | **Parameters*** | **Parameter Values** | **Range**^†^ | **Source** | |
| --- | --- | --- | --- | --- | --- | --- |
| *Outbreak-Related Expenses* |  |  |  |  |  | |
| Bottled water | Logistic Normal | μ (σ) | $72.85 ($82.39) | 0-∞ | Household survey | |
| Filter purchase | Gamma | α (β) | $1.12 ($118.87) | 0-∞ | Household survey | |
| Filter maintenance | Exponential | β | $89.58 | 0-∞ | Household survey | |
| Lodging outside Alamosa | Exponential | β | $332.13 | 0-∞ | Household survey | |
| *Direct Out-of-Pocket Health Care Costs* | |  |  |  |  | |
| Non-prescription drugs | Logistic Normal | μ (σ) | $35.87 ($35.97) | 0-∞ | Household survey | |
| Medical supplies | Logistic Normal | μ (σ) | $35.02 ($29.50) | 0-∞ | Household survey | |
| Doctor office visits | Exponential | β | $59.84 | 0-∞ | Household survey | |
| Prescription drugs | Gamma | α (β) | $2.03 ($7.92) | 0-∞ | Household survey | |
| Diagnostic tests | Exponential | β | $50.16 | 0-∞ | Household survey | |
| ER/hospital visits | Exponential | β | $117.11 | 0-∞ | Household survey | |
| *Indirect Costs of Acute Illness and Caretaking* | | | | | |  |
| Ill persons (full-time workers) | Gamma | α (β) | $ 2.35 ($150.34) | 0-∞ | Household survey | |
| Ill persons (part-time workers) | Gamma | α (β) | $ 2.35 ($75.17) | 0-∞ | Household survey | |
| Caretakers (full-time workers) | Logistic Normal | μ (σ) | $769.01($904.82) | 0-∞ | Household survey | |
| Caretakers (part-time workers) | Logistic Normal | μ (σ) | $ 384.51 ($452.41) | 0-∞ | Household survey | |
| *Alamosa Businesses* | Exponential | β | $11,377 | 0-∞ | Business Survey | |
| *Health Insurance Payment Costs* |  |  |  |  |  | |
| Doctor’s office visits | Logistic Normal**^‡^** | μ (σ) | $129 ($134) | 0-∞ | Local hospital | |
| Emergency room visit | Logistic Normal**^‡^** | μ (σ) | $693 ($874) | 0-∞ | Local hospital | |
| Hospitalization | Logistic Normal**^‡^** | μ (σ) | $7,011 ($6,241) | 0-∞ | Local hospital | |

^*^ μ (σ) refer to the Mean (SD) of a logistic normal, α (β) refer to the Alpha and beta of a gamma distribution and β refers to the beta of an exponential distribution.

^†^ 95 % of inputs in the simulations were within the maximum value recorded in the survey

‡ Assumed distribution, not derived through “Fit Distribution to Data” function

**Table S4: Comparison of household survey respondents to census data**

| **Characteristic** | **Alamosa Household Survey** | **2008 City of Alamosa Census** | **P** |
| --- | --- | --- | --- |
|  | (n=771) | (N=8,714 individuals, 3,302 households)^*^ |  |
| **Female** | **458/742 (62%)** | **4,430/8,714 (51%)** | **<0.001** |
| **Age in years (median)^†^** | **57 (n=724)** | **26.4** | **n/a** |
| **Race/ethnicity** |  |  | **<0.001** |
| Non-Hispanic white | 391/708 (55%) | 3,965/8,714 (46%) |  |
| Hispanic | 291/708 (41%) | 4,374/8,714 (50%) |  |
| **Income^‡^** |  |  | **<0.001** |
| <$13,000 (survey) or  <$15,000 (census) | 89/620 (14%) | 1,074/3,302 (33%) |  |
| $13,000-<$25,000 (survey) or  $15,000-<$25,000 (census) | 111/620 (18%) | 315/3,302 (10%) |  |
| $25,000-<$45,000 (survey) or  $25,000-<$50,000 (census) | 150/620 (24%) | 996/3,302 (30%) |  |
| $45,000-<$75,000 (survey) or  $50,000-<$75,000 (census) | 130/620 (21%) | 369/3,302 (11%) |  |
| >$75,000 (survey and census) | 140/620 (23%) | 548/3,302 (17%) |  |

^*^ Data source: Table DP05 2005-2009 American Community Survey 5-Year Estimates: Demographic and Housing Estimates (Accessible at: http://factfinder2.census.gov/bkmk/table/1.0/en/ACS/09_5YR/DP5YR5/1600000US0801090)

^†^ No statistical test conducted as Alamosa household survey respondents were required to be > 18 years of age

^‡^ Different income categories were used in the household survey and in the census; denominator for census is total number of households

**FIGURE S1. Schematic of the Monte Carlo Simulation Model for extrapolating costs to the City of Alamosa, Colorado**

Surveys of City of Alamosa households and businesses

Interview with local hospital

Census data

Eligible businesses in our convenience sample^*^

**ANALYSIS**

**RESULTS**

**VARIABLES**

**DATA SOURCES**

^*^ For businesses, we were only able to extrapolate to the total number of eligible businesses in our convenience sample (n=156), which was calculated by removing the number of undeliverable surveys (n=21) and surveys from businesses outside of Alamosa (n=5) from the total number of survey we distributed (n=182)

Results reported as **Total cost** which is the median of 10,000 iterations and **range** which represent the 5^th^ to 95^th^ percentile from the 10,000 iterations

Formula: **Total** X **Proportion** X **Cost distribution**

Monte Carlo simulation model using @Risk software (10,000 iterations)

Distribution of costs reported in surveys/

interviews

(See Appendix Table
S3 for details)

Proportion of individuals, ill persons, households or businesses that experienced a given cost

(See Tables 1, 6 and 8 in main text)

Total population, City of Alamosa, 2008 (N=8,746)

Total households, City of Alamosa, 2008 (N=3,302)

Total businesses potentially affected^*^ (N=156)
